# The use of a combined sliding skin graft and a free labial mucocutaneous graft for reconstruction of the equine upper eyelid after full‐thickness excision of a melanoma

**DOI:** 10.1002/ccr3.1992

**Published:** 2019-01-17

**Authors:** Andrea Steinmetz, Claudia Gittel, Denny Böttcher, Liv Lapko, Julia Offhaus

**Affiliations:** ^1^ Department of Small Animals Leipzig University Leipzig Germany; ^2^ University Equine Hospital Leipzig University Leipzig Germany; ^3^ Institute of Pathology Leipzig University Leipzig Germany

**Keywords:** eyelid melanoma, free labial mucocutaneous graft, horse, mucocutaneous junction, sliding skin graft

## Abstract

A melanoma of the upper eyelid was resected in a gray warmblood gelding. A full functional eyelid could be obtained by completion a sliding skin graft with a free labial mucocutaneous graft transplantation to restore the mucocutaneous junction and to decrease the risk of postoperative trichiasis.

## 1 INTRODUCTION

The most common equine periocular tumors are squamous cell carcinomas (SCC), sarcoids, melanomas, and lymphosarcomas.^1^ Nevertheless, melanoma is a relatively uncommon eyelid tumor in horses, because most of melanomas are found underneath the tail.^1^, ^2^ Horses with white or gray coat seem to be predisposed.^2^ Surgical excision is usually curative because most melanocytic eyelid masses in horses are benign, slow growing, and local.^3^ Only one report gave a description of the use of local photodynamic therapy following surgical resection.^4^ While using a full‐thickness excision to resect the tumor, the wound can be closed directly when the length of the removed eyelid marging is less than one‐third of the stretched eyelid length. If the tumor has a bigger size, the wound needs a blepharoplasty method to restore the eyelid length and function. An easy and widely used blepharoplasty technique is a H‐sliding skin graft plasty. We prefer to use the term sliding skin graft. Nevertheless, this technique restores the eyelid margin but includes the postoperative risk of trichiasis. In this clinical case report, we show the combination of a sliding skin graft with a free labial mucocutaneous graft to overcome this disadvantage. By bringing in the free labial mucocutaneous graft, we created a new hair‐free lid margin.

## 2 CASE HISTORY AND CLINICAL EXAMINATION

A 9‐year‐old, 550 kg, gray warmblood gelding was presented for evaluation of a mass protruding from the left dorsal eyelid and causing moderate discomfort and discharge. The mass had been noticed by the owner 4 weeks prior to the presentation.

During the physical examination, a second‐degree atrioventricular‐block was noticed. This can be considered as a physiological finding due to an elevated vagal tonus in trained horses and was without further consequences. Apart from the above and the ophthalmologic symptoms, no other abnormality was registered and there was no evidence of other cutaneous or subcutaneous masses. The serum chemistry and cell blood count were unremarkable.

**Figure 1:**
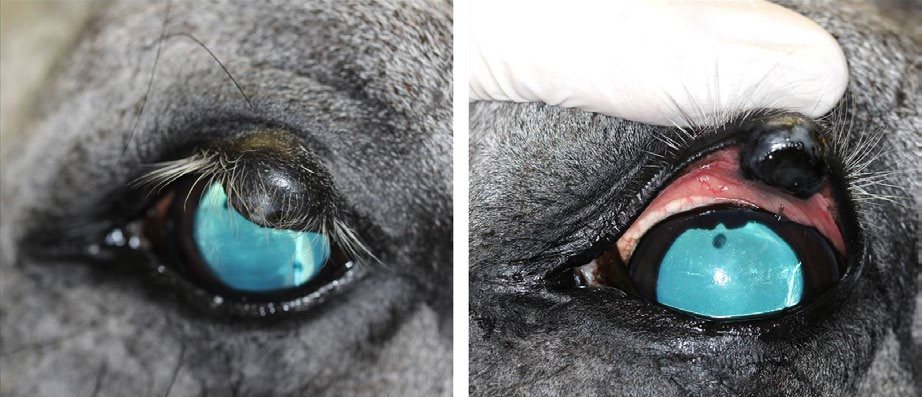
Dark pigmented mass in the left upper eyelid

The ophthalmological examination revealed moderate blepharospasm with ocular discharge and a heavily pigmented mass on the upper left eyelid. Direct and consensual pupillary light reflexes (PLR), palpebral reflexes, the menace responses, and dazzle reflexes were normal for both eyes. After sedation with 10 μg/kg detomidine hydrochloride (Cepesedan RP 10 mg/mL; CP‐Pharma, Burgdorf, Germany), intravenously (IV) the slit‐lamp biomicroscopy (SL 14; Kowa company, Tokyo, Japan) revealed a locally restricted, dark pigmented mass, about 13 × 10 × 8 mm in diameter, with a rough opening on the conjunctival side, about 3 mm in diameter in the left upper eyelid (Figure 1). Subsequent to the mass in the upper eyelid, an irregular, round superficial central corneal ulceration, 1 cm in diameter, without stromal loss, infiltration, or neovascularization, and corresponding to the rough surface of the tumor was detected. No abnormalities were observed in the remaining ocular structures of the left eye and in the complete right eye on slit‐lamp biomicroscopy and direct ophthalmoscopic examination (Welch Allyn Ophthalmoscope™, Hechingen, Germany). The intraocular pressures of both eyes were within normal limits (21 mm Hg in the left and 20 mm Hg right eye) as measured using applanation tonometry (Tono‐Pen Vet™; Eickemeyer, Tuttlingen, Germany). Ultrasonography of the mass (linear probe 10 mHz; My Lab™ EightVet; Esaote Biomedica, Köln, Germany) showed a homogeneous isoechogenic structure starting from the eyelid margin and infiltrating to the eyelid tarsus 2 × 1.5 cm in diameter. A melanoma of the upper eyelid was suspected and surgical removal was planned. Further diagnostics for staging like lymph node fine needle aspiration were declined by the owner.

### 2.1 Surgical treatment

Prior to surgery amoxicillin‐natrium 10 mg/kg IV (Amoxisel^®^; Selectavet Dr. Otto Fischer GmbH, Weyarn‐Holzolling, Germany), gentamicin 6.6 mg/kg IV (Genta^®^ 100 mg/mL; CP‐Pharma Handelsgesellschaft mbH) and a nonsteroidal anti‐inflammatory drug, flunixin meglumine 1.1 mg/kg IV (Flunidol^®^ rps 50 mg/mL; CP‐Pharma Handelsgesellschaft mbH), were administered for peri‐ and postoperative antibiotics and analgesics. The premedication consisted of acepromazine 0.03 mg/kg IV (Vetranquil^®^ 1%; Ceva Tiergesundheit GmbH, Düsseldorf, Germany), romifidine 0.06 mg/kg IV (Sedivet^®^, Boehringer Ingelheim Pharma GmbH & Co. KG, Ingelheim am Rhein, Germany), and l‐methadon in a fixed combination with fenpipramide 0.05 mg/kg IV (l‐Polamivet^®^, Intervet Deutschland GmbH, Unterschleißheim, Germany). General anesthesia was induced with diazepam 0.08 mg/kg IV (Ziapam^®^; Ecuphar GmbH, Greifswald, Germany) and ketamine 2.8 mg/kg IV (Ursotamin^®^; Serumwerk Bernburg AG, Bernburg, Germany). During the surgery in right lateral recumbency, anesthesia was maintained with isoflurane (Isofloran CP^®^; CP‐Pharma) in oxygen in combination with a romifidine continuous rate infusion of 0.06 mg/kg/h. Standard fluid administration and routine monitoring was performed throughout anesthesia.

The surgical technique of a sliding skin graft or advancement flap^5^, ^6^ was elected. The left eyelid, the eye, and the left lips were prepared and flushed with betadine.

**Figure 2:**
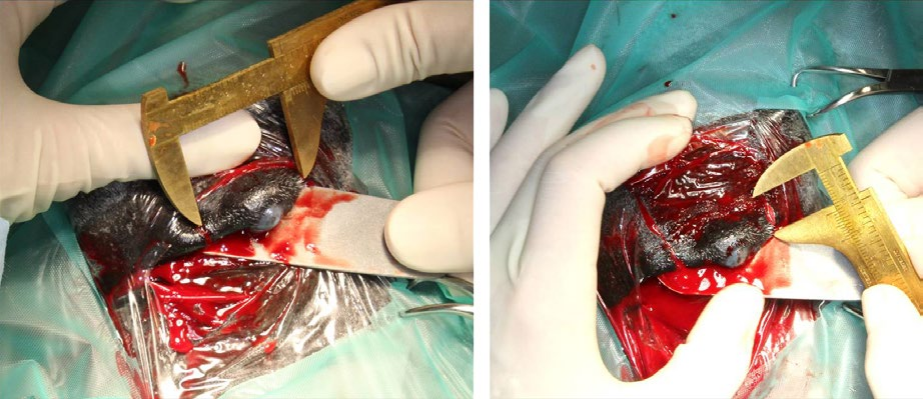
Measurement of the tumor size and the length of the upper eyelid which had to be removed

A Gränitz eyelid spatula™ (Eickemeyer) was used to stabilize the upper eyelid and protect the cornea. Initially, two full‐thickness incisions perpendicular to the free eyelid margin and about 5 mm apart from the visible mass were made. The third full‐thickness incision was parallel to the lid margin and connected the proximal ends of the first two incisions. The excised tissue measured about 30 mm of the free lid margin and about 20 mm of the skin of the upper eyelid (Figure 2). The resected tissue was fixed in formalin and submitted for histopathologic examination. The initial perpendicular incisions were elongated proximally with about five centimeters. The skin‐flap was mobilised without problems using blunt and sharp dissection.

**Figure 3:**
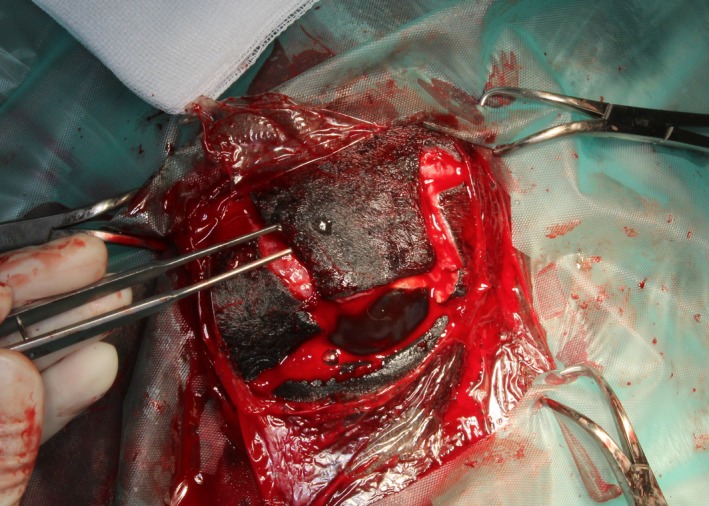
Two Burrow triangles at the flap‐base were removed

**Figure 4:**
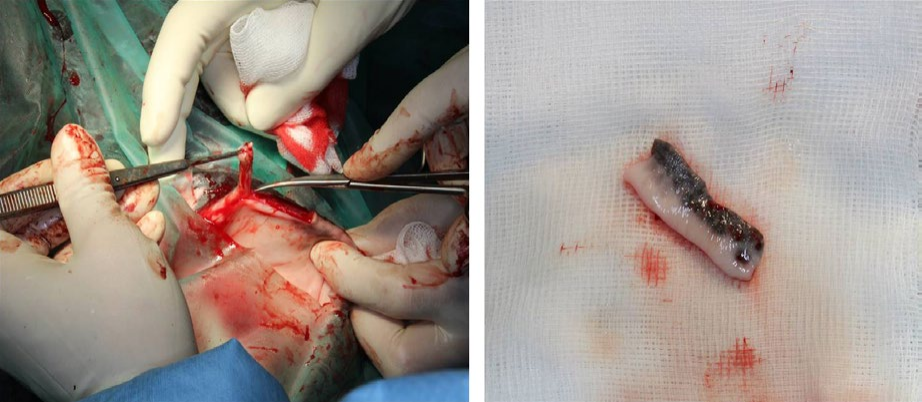
Harvesting of a free labial mucocutaneous graft

**Figure 5:**
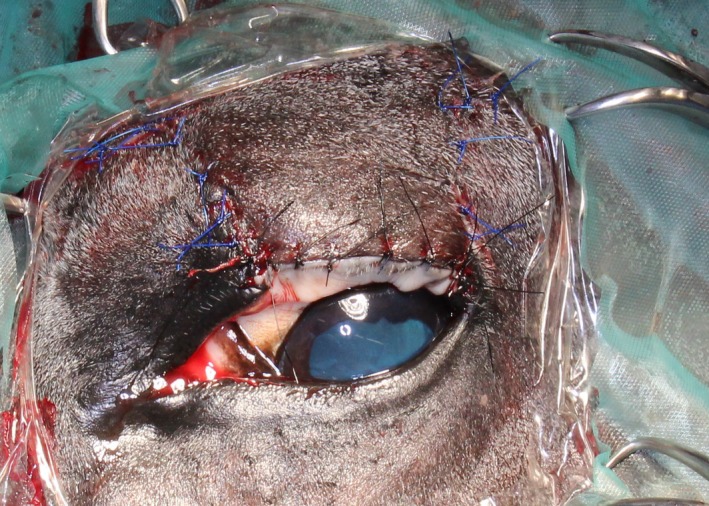
Free labial mucocutaneous graft sutured with the sliding skin graft

**Figure 6:**
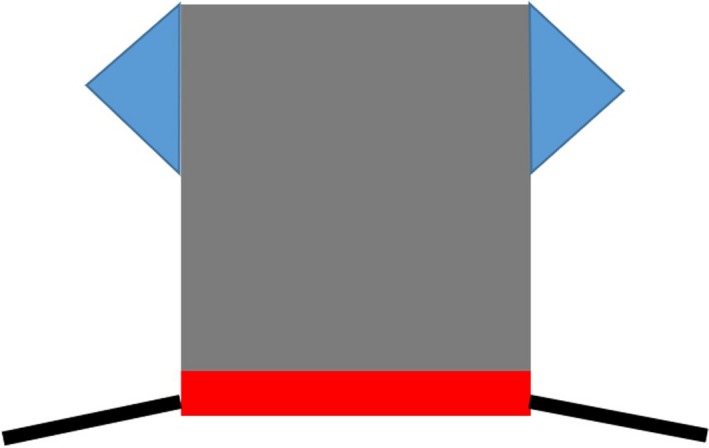
Procedure drawing: sliding skin graft, gray; Burrow triangles, blue; free labial mucocutaneous graft, red; eyelid margin, black

Subsequently, two small Burrows triangles and approximately 5 mm of the free wound margin were removed to improve the apposition of the skin (Figure 3). In the next step, the skin flap was retracted distally to fill nearly the complete defect. The flap was sutured subcutaneously in continuous manner with absorbable material (4/0 Monocryl^®^; Ethicon, Hamburg, Germany). The skin was sutured with nonabsorbable material (3/0 Ethilon^®^; Ethicon) in an interrupted pattern. In the next step, about 30 mm of the mucocutaneous junction of the lower lip was harvested (Figure 4). The wound from the lower lip was closed with simple interrupted sutures and absorbable material (3/0 Monocryl^®^; Ethicon). The graft was about as long as the excised part of the eyelid, and the mucosa side of the lip margin was about two‐third of the width of the graft. The free graft was transplanted in the distal part of the skin flap to replace the eyelid margin. The oral submucosa of the transplant was attached to the subconjunctival continuously by a mattress suture with absorbable material (4/0 Monocryl^®^; Ethicon). Care was taken to ensure that no suture material overhung the surface of the conjunctiva. To append the eyelid skin with the skin part of the labial graft, interrupted sutures with nonabsorbable material were used (4/0 nylon; Ethilon^®^; Ethicon; Figures 5 and 6). A partial medial temporary tarsorrhaphy was made and a subpalpebral lavage system (Equivet; Kruuse, Langeskov, Denmark) was placed ventrotemporal to facilitate local postoperative medications.

Subsequently, a contact lens (Acrivet Pat H1; Bausch&Lomb, Greenville, SC, USA) was used to protect the corneal surface and allow the healing of the corneal ulceration. To ensure further protection of the eye, the head was bandaged for 8 days. The bandage was changed every 2 days.

### 2.2 Histopathologic examination

The sample was 32 × 23 × 9 mm in size and showed a well‐defined dark brown mass measuring 11 × 11 × 9 mm.

**Figure 7:**
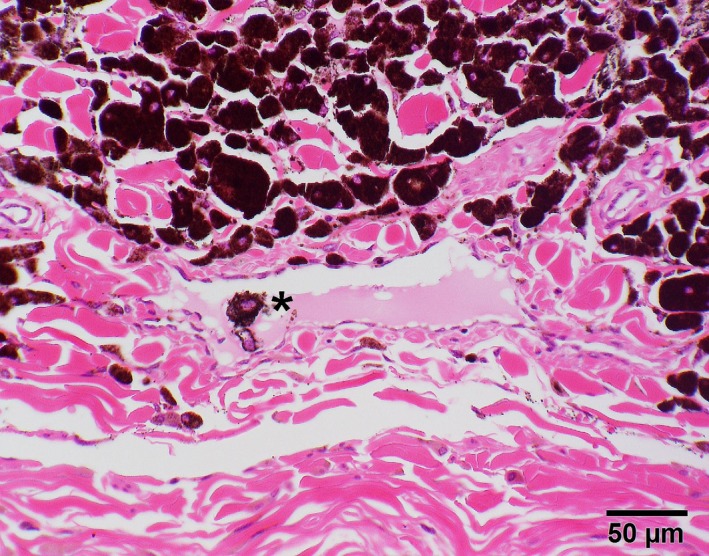
Photomicrograph of dermal mass consisting of heavily pigmented neoplastic cells occasionally showing invasion of lymphatic vessels (asterisk). HE stain

**Figure 8:**
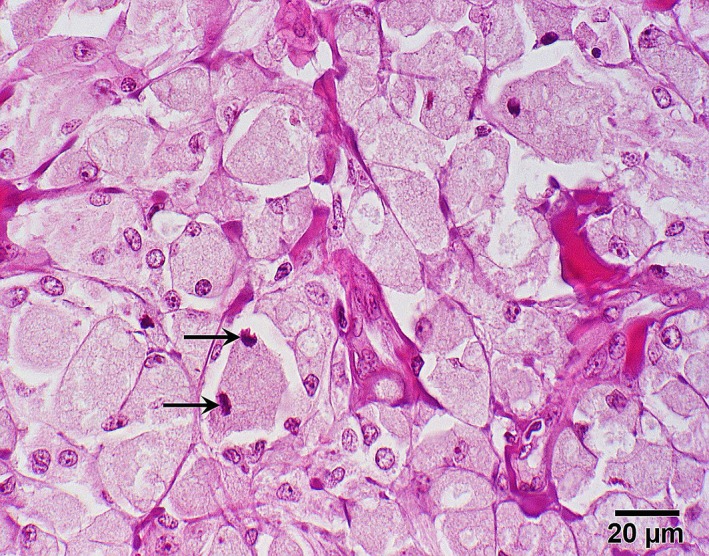
Photomicrograph of neoplastic tissue. Bleaching of intracellular pigment in neoplastic cells reveals mitotic figures (arrows). Hydrogen peroxide bleaching followed by HE stain

Microscopically, this mass presented as a nonencapsulated but well‐demarcated dermal tumor mainly being comprised of highly pigmented cells. Most of these were round (to polygonal), had discrete borders and prominent dark brown coarse intracytoplasmic pigment (Figure 7), which could be effectively bleached using hydrogen peroxide (Figure 8). Nuclei showed one to two small nucleoli; mitotic figures were sparse with 1‐2/HPF (Figure 8). Occasionally, an invasion of neoplastic cells into lymphatic vessels could be observed (Figure 7). Altogether, the evaluated histopathologic findings indicated a malignant neoplasia of melanocytic origin. Within the microscopically examined sections, there were no findings suggesting incomplete surgical removal.

### 2.3 Outcome and follow‐up

For postsurgical antibiotic treatment, amoxicillin 10 mg/kg q12h IV (Amoxisel 100 mg/mL; Selectavet) and gentamicin 6.6 mg/kg q24h IV (Genta 100 mg/mL; cp‐parma) were administered systemically for 5 days. Furthermore, flunixin meglumine was given as anti‐inflammatory medication 1.1 mg/kg q24h IV (Flunidol RPS 50 mg; CP‐Pharma) for a period of 7 days.

The topical treatment consisted of 0.2 mL tobramycin (Tobramaxin^®^ 3 mg/mL; Alcon, Freiburg, Germany), 0.1 mL voriconazole 1% (VFEND^®^ 200 mg; Pfizer, Freiburg, Germany), 0.1 mL autologous serum, 0.1 mL EDTA (1.6 mg K3 EDTA/mL), and 0.1 mL atropine sulfate (Atropin‐POS 0.5%^®^; Ursapharm Arzneimittel GmbH, Saarbrücken, Germany). These ophthalmic solutions were applied one after the other every 2 hours for 11 days.

Four days postoperatively, the temporary tarsorrhaphy suture was removed. At this time, the graft seemed to be viable. The superficial corneal ulceration healed in 7 days and the bandage contact lens was removed. Two weeks postoperative, a small lateral part of the free labial mucocutaneous graft had become necrotic and was replaced by healthy granulation tissue.

**Figure 9:**
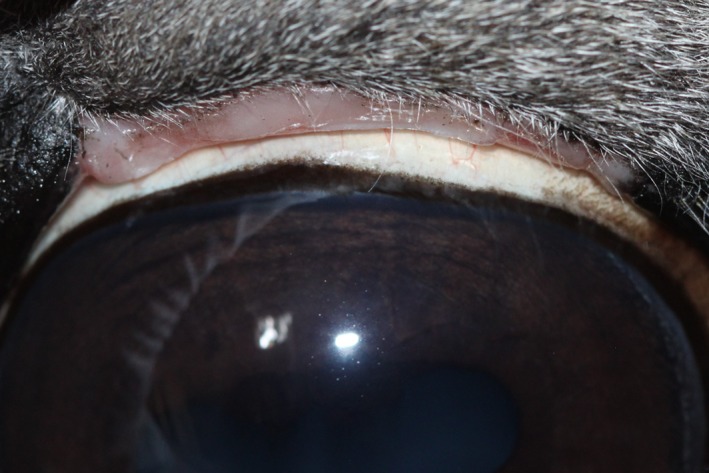
Artificial hairless lid margin 7 wk postoperatively

**Figure 10:**
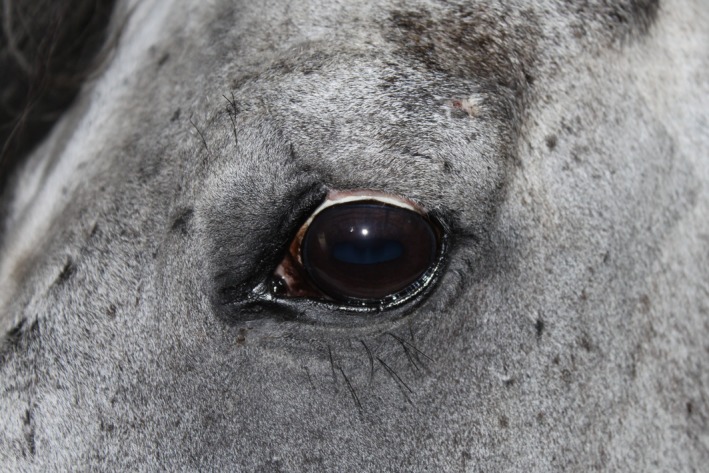
Excellent cosmetical result 7 wk postoperatively

Seven weeks postoperative, the eye was without irritation and showed a hairless mucocutaneous eyelid margin (Figure 9). The overall cosmetic result was excellent (Figure 10). The lip‐defect healed without complications. Follow‐up by telephone revealed no regrowth of the tumor up to 6 months postoperatively.

## 3 DISCUSSION

Melanomas occur more common under the tail, the perianal region, and the lips than in the periocular tissue^2^ On the other hand, from the equine melanomas involving the eye the ones involving the periocular skin are the most common.^3^

Horses could have dermal melanomas for years (range 1‐6 years) before succumbing to metastatic disease. The histologic characteristic of dermal masses is not predictive of malignancy in the majority of cases. In this case, an invasion of neoplastic cells into several lymphatic vessels was obvious which may lead to metastasis to regional lymph nodes as well as to distant sites.^7^ Unfortunately, further diagnostics for staging like lymph node fine needle aspiration were declined by the owners. Otherwise the gelding was presented in the early stage of tumor growing and showed no abnormalities in the further clinical examination. All palpable lymph nodes showed normal size and structure and were repeatedly examined. The tumor was completely resected and the horse showed no signs of local regrowth or metastatic disease 12 months postoperatively.

Described treatment modalities for melanomas in horses are surgical excision, administration of cimetidine, autogenous vaccine, surgical debulking, and additional phototherapy.^4^, ^8^, ^9^ Oral cimetidine has been shown to shrink nonocular melanocytic tumors in horses, but its efficacy has not been reported in the management of eyelid melanocytic tumors in this species.^8^ In one report, no significant clinical regression in the tumor size of the eyelid melanoma was noted after a 6 months oral cimetidine therapy and the experimental use of local photodynamic therapy following surgical resection of an equine melanocytic tumor was performed. No evidence of regrowth was present within the follow‐up of 60 days.^4^ Other adjunctive therapy modalities such as cryotherapy, intralesional chemotherapy and immunotherapy, radiation and cytotoxic therapy have not been reported with equine melanocytic tumors.

In this case, reconstructive surgery was chosen to prevent secondary healing and to obtain an excellent eyelid conformation and function. The integrity of the eyelids is important for the tear film maintenance and distribution, protection of the cornea, and thus to preserve the eye and the vision.^1^ Wilkie^10^ used a silicon implant to cover a defect in the upper eyelid of a horse because surgical reconstruction of the eyelids after the resection of a greater tumor mass is challenging in horses because of the tight junction of the skin to the underlying bone and fascia. Other described reconstructive blepharoplastic procedures in horses are sliding skin graft or H‐plasty, tarsoconjunctival advancement graft, pedicle skin graft, full‐thickness eyelid graft, rhomboid graft, sliding Z‐graft, and various combinations.^5^, ^11^ Sliding skin graft or H‐plasty is one described reconstructive technique after resection of a melanoma in the upper and lower equine eyelid.^9^, ^12^ In this study, the authors preferred to use the term “sliding skin graft” because H‐plasty in reality is a double sliding skin graft. Reconstruction of a mucocutaneous junction of the eyelid in horses has not been reported yet. Nevertheless, a hairless eyelid margin is important to decrease the risk of postoperative trichiasis. In small animals, lip to lid transposition flaps have been performed to reconstruct the eyelid margin.^13^, ^14^ This technique cannot be used in horses because of the great distance between the lip and the eyelid. The recently described free tarsomarginal autograft in dogs^15^ would attain a deformed lid cleft in horses. Some reconstruction of an eyelid margin can be attained by using the tarsoconjunctival advancement graft. Nevertheless, this is a two‐step procedure^5^ and the eye is blind during the first healing phase of about 2 weeks.

In the case of this study, it was decided to remove the tumor, create a sliding skin graft, and shape a hairless eyelid margin by using a free labial mucocutaneous graft transplantation in one session. Because of complexity of the surgery, the procedure took place in general anesthesia like recommended in literature for greater defects.^16^

In the present case, the lavage system was placed through the ventral eyelid to minimize possible complications^17^). Chen (2010) reported a tear volume 233.74 μL in eyes of healthy horses. Because of the complete recycling of the tear volume in approximately 7 minutes, he suggested increasing dosing regimens or constant infusion methods for treating corneal diseases in horses.^18^ In the present case, high‐frequency administration of four different drugs was used to reach high ocular surface concentration because the humidity under the head bandage could impair the healing of the corneal ulcer.

Survival of free grafts depends on early reestablishment of sufficient circulation to provide nutrition and to discharge metabolic waste products. Regeneration initially progresses more slowly than degeneration. New capillary ingrowth occurs at approximately 0.5 mm/d. For this reason, a risk of ischemic necrosis of the graft is given until the vascularization is completed.^19^ In this case, a small part of the free lip graft had become necrotic. Nevertheless, the remaining tissue rebuilt a new hairless mucocutaneous junction. The follow‐up by telephone 12 months postoperative revealed that the eyelid was full functional. The eye showed no irritation, the cosmetic result was excellent, and no regrowth of the tumor could be seen.

## 4 CONCLUSION

Reconstruction of a fully functional eyelid can be obtained in horses by completion a sliding skin graft with a free labial mucocutaneous graft transplantation to restore the mucocutaneous junction after resection of malignant tumors of the upper eyelid.

## CONFLICT OF INTEREST

There is no conflict of interest.

## AUTHOR CONTRIBUTIONS

AS: Acted as main surgeon, main author, written the most parts, and composed the paper and figures. CG: Acted as anesthetist, and written the parts general clinical examination and anesthesia. DB and LL: Involved in histopathologic examination of the tumor and writing of this part and figures. JO: Assisted the operation, and written the postsurgical treatment part and figures.
